# Merging Preclinical EPR Tomography with other Imaging Techniques

**DOI:** 10.1007/s12013-019-00880-7

**Published:** 2019-08-22

**Authors:** Michal Gonet, Boris Epel, Howard J. Halpern, Martyna Elas

**Affiliations:** 1 Faculty of Biochemistry, Biophysics and Biotechnology, Jagiellonian University, Krakow, Poland; 2 Radiation and Cellular Oncology, University of Chicago Pritzker School of Medicine, and Center for Electron Paramagnetic Resonance Imaging In Vivo Physiology, University of Chicago, Chicago, IL USA

## Abstract

This paper presents a survey of electron paramagnetic resonance (EPR) image registration. Image registration is the process of overlaying images (two or more) of the same scene taken at different times, from different viewpoints and/or different techniques. EPR-imaging (EPRI) techniques belong to the functional-imaging modalities and therefore suffer from a lack of anatomical reference which is mandatory in preclinical imaging. For this reason, it is necessary to merging EPR images with other modalities which allow for obtaining anatomy images. Methodological analysis and review of the literature were done, providing a summary for developing a good foundation for research study in this field which is crucial in understanding the existing levels of knowledge. Out of these considerations, the aim of this paper is to enhance the scientific community’s understanding of the current status of research in EPR preclinical image registration and also communicate to them the contribution of this research in the field of image processing.

## Introduction

The application of electron paramagnetic resonance (EPR) spectroscopy and imaging to human subjects has been made possible by recent technical and scientific developments. The wide range of electron paramagnetic resonance-imaging (EPRI) applications enabling mapping live tissue microenvironment parameters such as redox state, pH, thiol concentration, and especially oxygen partial pressure (pO_2_). This makes EPRI an attractive functional-imaging modality for medical applications. However, insufficient anatomical details provided by EPRI, necessitates the use of other anatomic-imaging references for accurate interpretation of the EPR images, especially in an in vivo setting. The question arises how to merge EPRI with tissue structure-imaging modalities. This is important in the context of future human applications and preclinical studies.

Modern EPR tomography allows for imaging of laboratory animals with excellent spatial and temporal resolution [1–5]. Recent works demonstrate the feasibility of EPRI to study redox status [6], pH [7], thiol concentration [8, 9], and oxygenation [8, 9] in living animals.

Anatomic reference can be provided by nuclear magnetic resonance imaging (MRI), X-ray computed tomographic imaging (CT), or ultrasound imager. Several different approaches to correlate anatomical information with EPRI have been developed over the years. These include: building dual-modality imagers where both MRI and EPRI is performed on the same animal [10], applying image registration procedure [11], choosing anatomically referenced region of interest (ROI) by using loop resonator [12], modulated field gradient EPRI [13] allowing to image only a chosen part of the body, and overlaying EPR images with anatomical atlas. For ex vivo measurements such as bioptates, excisions, and histological sections, photographic images of the samples can be used [14–16]. All of the above solutions allow for merging functional EPR images with an anatomical reference.

## How to Support EPR Imaging

**Fig. 1:**
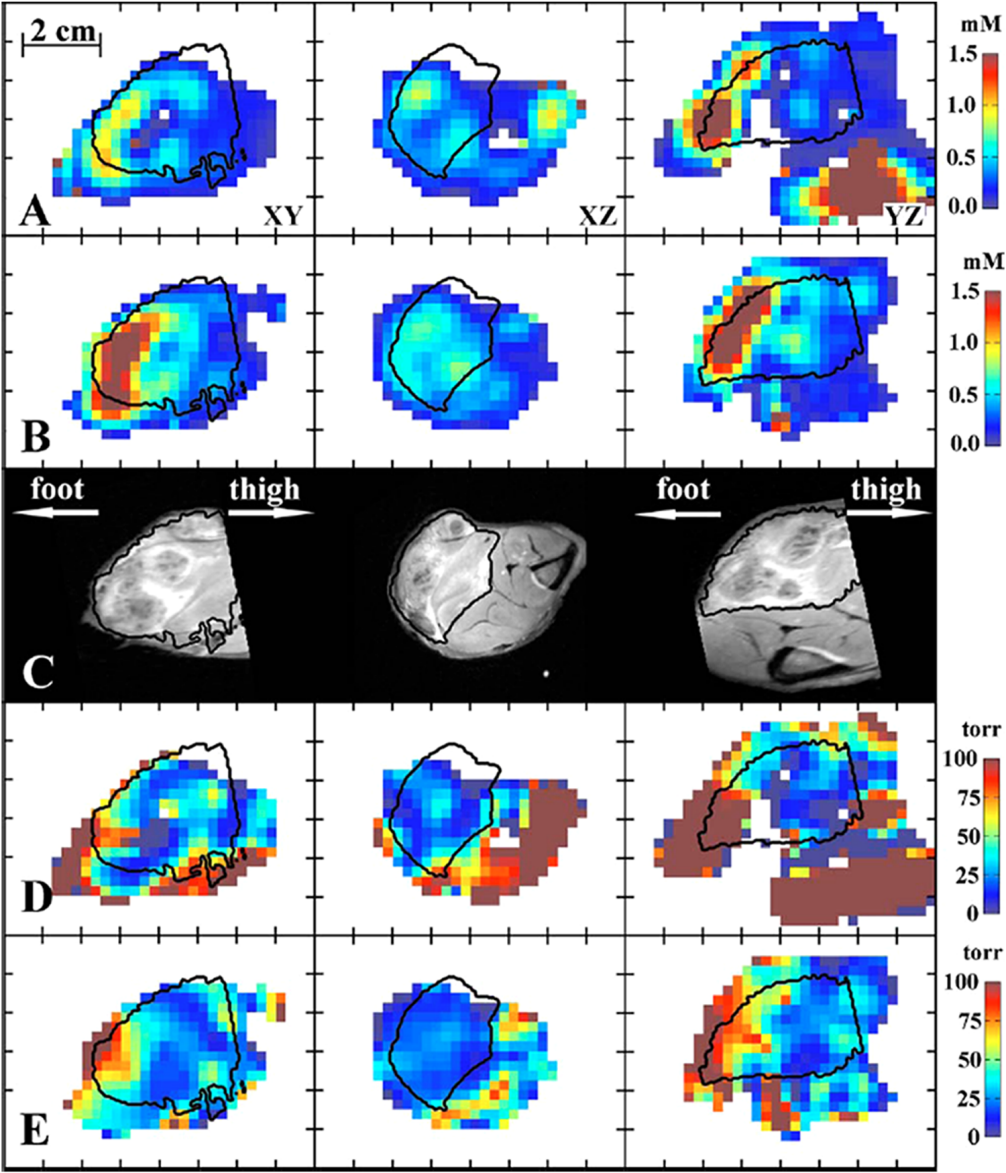
Selected slices of images: **a** CW concentration, **b** ESE concentration, **c***T*_2_ weighted MRI, **d** CW pO_2_, and **e** ESE pO_2_. The tumor region is determined from the MRI image and is outlined by contours (adopted from [34])

More and more aspects of tissue functioning, normal, and pathological, can be studied using EPR and EPRI. But an accurate interpretation of the results is often possible only if a complete information, both functional and structural, is acquired from the imaged organism. EPRI alone however does not provide a sufficient anatomical reference of the imaged object. Even though current imagers allow to obtain high spatial resolution images, they still only provide information about the volume in which spin probe is distributed, without accurate tissue borders or discernment between different tissues. For example, during tumor imaging the distribution of the spin probe is not very different between tumor and muscle in EPRI spatial images, while in MRI the tissue structures are clearly visible (Fig. 1).

Therefore, complementary techniques for anatomical reference need to be implemented together with EPRI. To meet this challenge the most often used solutions are: (i) developing hybrid systems for simultaneous imaging using both nuclear and electron magnetic resonance without having to move the animal between imagers; or (ii) image co-registration, which allows for fusion of the information from different images of the same object, regardless of their modality. Both approaches have opened a new opportunity window for biomedical research; finally, (iii) even if neither of above may be applied, there is a possibility of implying internal anatomy or structure from the a priori knowledge.

### EPRI–MRI Hybrid Systems and PEDRI

**Fig. 2:**
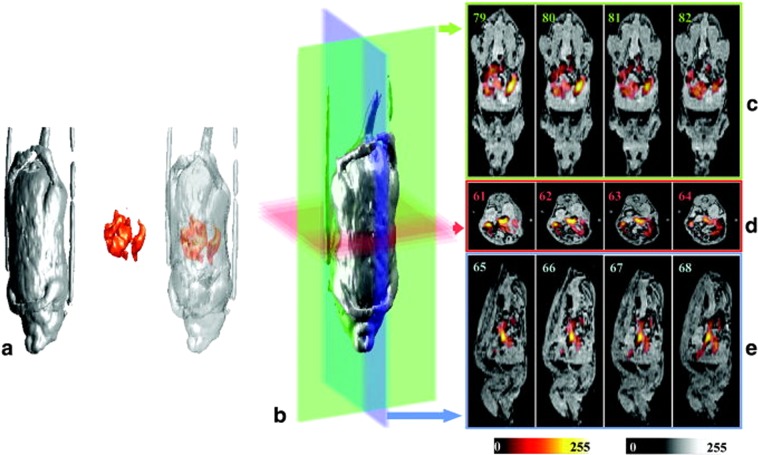
EPR/MRI fused images provide by hybrid EPR/MRI system. **a** 3D renderings of the proton MRI, EPRI, and fused images from a live mouse fed paramagnetic charcoal. **b** 3D rendering of the MR image dataset showing the planes for the slices depicted in **c**–**e**. Colors of the planes correspond to the frames containing the slices shown: **c** coronal slices, **d** axial slices, and **e** sagittal slices of the fused images. Color scale: MRI, grayscale; EPRI, hot metal scale (adopted from Samouilov et al. [19])

Similar physical and technical foundation of the methods stimulated inventors towards developing hybrid systems that allow to conduct MRI and EPRI experiment in the same device. Such approach was first reported by the Zweier group [17] at 750 MHz for EPR and 16.18 MHz for MRI. Subsequently, Matsumoto et al. [18] reported co-imaging of mice with EPRI at 300 MHz and proton MRI at 8.5 MHz. In 2007 Samouilov et al. developed hybrid EPR/NMR co-imaging system where EPRI was performed at ca. 1.2 GHz (L-band) and MRI at 16.18 MHz made the hybrid systems suitable for whole-body co-imaging of living mice [19]. In 2010 the same group built a dual-frequency resonator for EPR/MRI co-imaging, to avoid errors in EPR and MRI co-imaging registration caused by inadvertent animal movement [20]. Recently, several studies have been published demonstrating that the hybrid approach has become a mature technique, bringing novel information in various settings. For example, Caia et al. have applied EPR/MRI co-imaging system for the mapping of in vivo redox state both in control and cigarette smoke-exposed mice [21]. Another approach combining MRI end EPR was to employ EPR-related microwave irradiation with low-field magnetic resonance imaging so that the electron spin polarization is transferred to nearby protons, resulting in stronger signals. This approach is called proton-electron double-resonance imaging (PEDRI), also known as Overhauser MRI (OMRI) or a liquid-state dynamic nuclear polarization (DNP MRI). Imaging of PEDRI is effectively an indirect form of EPRI in the sense that enhancement of proton signals is proportional to the concentration of spins in the paramagnetic system and the degree of saturation of the EPR system. In PEDRI image, regions of the sample which containing free radicals have much higher intensity due to the Overhauser-enhanced proton nuclear magnetic resonance signal, revealing the location of the free radicals [22–24]. A significant impact in this field was given by Prof. Lurie and Prof. Zweier groups, by developing PEDRI of free radicals using a clinical MRI system [10] and then upgrading it successfully into the co-registered system. This approach allowed for the MRI/EPR image registration of living mice, so that the whole procedure of setup and imaging took ~40 min [17, 19]. This way EPRI has been linked with MRI, and thus, functional images of oxygenation or redox state obtained by EPR have been fused with high-resolution anatomical images provided by MRI. Li et al. used a PEDRI system for rapid imaging and evaluation of the in vivo distribution and clearance of triarylmethyl radical in mice [25]. In 2014 Samouilov et al. have used an advanced nitroxide probe to image extracellular tumor pH using double-resonance imaging, providing an analytical approach for spatially resolved noninvasive pH monitoring, in vivo [26]. An exhaustive review about in vivo application of PEDRI was delivered by Kishimoto et al. [27]. In Table 1 significant studies where hybrid systems EPR/NMR and PEDRI were used for in vivo applications are displayed and an example of in vivo application of the hybrid system, showing the distribution of charcoal in the body of a mouse is shown in Fig. 2 [19].

**Table 1:** Examples of studies applying NMR/EPR hybrid or PEDRI systems for in vivo preclinical studies

Authors	EPR imaging	Spin probe	Supported technique	System	In vivo application
Krishna et al. [40]	3D (spin probe distribution) and 4D (pO_2_ maps)	Ox063	MRI	PEDRI	Mouse whole body, tumor
Lurie et al. [10]	2D (spin probe distribution)	TAM	MRI	PEDRI	Mouse whole body
Li et al. [25]	2D (spin probe distribution)	TAM	MRI	PEDRI	Mouse whole body
Li et al. [41]	2D (spin probe distribution)	PCA^a^, 3-CP, TEMPONE^b^	MRI	PEDRI	Mouse whole body
Utsumi et al. [42]	2D (spin probe distribution and pharmacokinetics of spin probe)	15N-oxo-TEMPO, 14N-carbamoyl-PROXYL	MRI	PEDRI	Mouse whole body
Matsumoto et al. [43]	2D (spin probe distribution)	3-CP	MRI	PEDRI	Mouse kidney
Samouilov et al. [19]	3D (spin probe distribution)	paramagnetic charcoal	MRI	Hybrid	Mouse whole body
Matsumoto et al. [44]	2DS (pO_2_ maps)	Ox063	DCE-MRI	PEDRI	Tumor
Ahmad et al. [45]	3D (spin probe distribution)	LiPc	MRI	Hybrid	Mouse whole body
Caia et al. [21]	3D (spin probe distribution)	3-CP	MRI	Hybrid	Mouse whole body
Krishna et al. [46]	(pO_2_ maps)	Ox063	MRI	PEDRI	Mouse whole body, tumor
Samouilov et al. [26]	2D (pH maps)	Im6^c^	MRI	PEDRI	Mouse mammary gland

^a^2,2,5,5-Tetramethyl-3-carboxylpyrrolidine-N-oxyl

^b^2,2,6,6-tetramethyl-4-ox-opiperidine-N-oxyl

^c^2-(4-((2-(4-Amino-4-carboxybutanamido)-3-(carboxymethylamino)-3-oxoproylthio)-methyl)phenyl)-4-pyrrolidino-2,5,5-triethyl-2,5-dihydro-1Himidazol-1-oxyl-D11

EPR/MRI hybrid imagers and PEDRI systems make it possible to obtain both anatomical and functional images provided by these two magnetic resonance techniques without moving the specimen. The benefits of such solution fulfill the requirement of anatomical reference for EPRI maps from a single experiment in relatively short timescales, without changing the position of the imaged animal. So far, however, this approach is limited to two magnetic resonance methods. To involve more imaging techniques where physical basics are not as similar as EPR/MRI, such as CT, PET, SPECT or ultrasound, a correct fusion of multiple images from different methods has to be performed. The process of accurate overlapping of the images from separate imaging techniques is referred to as image registration and was initially developed for clinical applications [28, 29].

### Image Registration

Image registration is the process of finding correspondence between all points in at least two images of the same object [11]. Images may be two or more dimensional, be obtained from different modalities, have different resolutions and orientations. For the images which do not possess sufficient common information, like in the case of EPR and MRI images, artificially added common points of reference, typically referred to as fiducials, are used. A key element in image registration lies in the proper selection of fiducials markers. Fiducials markers are usually rigid objects placed in the imaging field of view in such way that they are fixed in relation to the imaging region. Four nonplanar points in space which do not belong to any symmetry group establish a unique definition of linear coordinate system which for registration procedure ensures the unambiguous location. Moreover, these markers have to be visible in all acquired images from each modality used. In the case of EPRI, thin tubes filled with the radical-containing solution can be used. For registration reference, the radical should exhibit a single, narrow EPR line, which is resulting in high-quality EPR images. For MRI a similar approach can be used with fiducials filled with water. Fiducials filled with a low-concentration radical solution can be visible both in EPRI and MRI. Other modalities as CT or micro-CT typically use heavy-metal containing markers, however, glass tubes can also give sufficient contrast. Thus, markers seen by EPRI, MRI, and CT may consist of thin glass tubes placed close to the imaged object, filled with aqueous solution of a paramagnetic spin probe.

The fixing of the fiducials to the imaged object and ensuring the same position in two (or more) imaging modalities is completed by two methods: (i) building a single-use cast or a mold using a non-paramagnetic material which immobilizes the imaged object and the fiducials; (ii) designing a multiple-use cradle or bed for the animal, with built-in position for fiducials. Both approaches have their advantages and limitations. Building a cast is useful when only a part of the animal body is going to be imaged, e.g., the leg with the tumor. The custom cast for the animal may be prepared very quickly, in such way that it adequately fills the resonator and allows positioning the object ROI and the fiducials in the center of the resonator. When the imaging device changes, an animal with the cast intact is moved between instruments. The second approach, using a special bed or cradle-containing fiducials adapted to hold animals may be more advantageous for a whole-body experiment. If the cradle is compatible with all imaging instruments, this approach might be better for the animal.

**Fig. 3:**
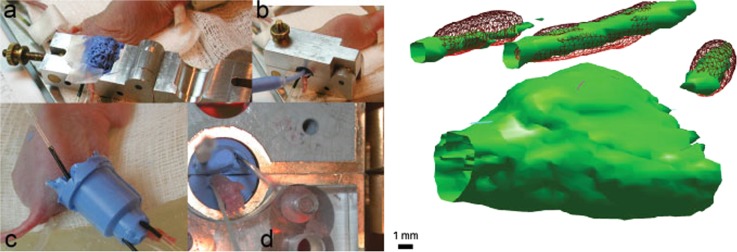
**a**–**d** (Left) Mouse leg cast with fiducials for EPR/MRI registration. Right: An example of image registration used in MRI and EXPI experiment. Right: 3D surface from an MRI spin echo is in green and registered EPRI fiducials are in red mesh (see ref. [30])

A crucial role in the preclinical EPR-imaging research, where anatomy images were obtained using MRI, was played by Halpern’s group. Using vinyl polysiloxane cast material or 3D-printed animal cradles Halpern’s group had a lot of success in the correlation of in vivo pO_2_ images from mice, rats, and rabbits not only with MR images but also with CT and PET modalities. Vinyl polysiloxane dental material was shown to be a convenient material for immobilization of animal and fiducials close to an animal leg. It has been successfully used to multimodality-imaging combining EPRI with MRI, (Fig. 3, [30]). Having established the procedure of registration of the EPRI with MRI, in all the subsequent studies, EPRI hypoxia maps of tumors were supported by accurate tumor anatomy images obtained from MRI. For example, they have demonstrated that EPR image hypoxic fraction separates the population of fibrosarcomas that are cured from those that fail after a single radiation dose in in vivo model [31]. Similarly, it was shown that tumor hypoxia measured by EPRI is highly predictive for their response to radiotherapy. Data showed that low hypoxic tumors treated to nominal TCD_50_ were ca. three times more controlled than poorer oxygenated tumors [32]. Haney et al. reported that a quantitative absolute oxygen measurement correlated with tissue perfusion as determined by DCE MRI, in vivo [33]. This image-guided approach for antivascular therapy may help to identify regions of good and poor response for treatment [33]. In 2010 Epel et al. presented methodologies for EPR/MRI image registration in oxygen imaging in the rabbit leg bearing a VX-2 tumor (Fig. 1) [34]. This study paved the way for the application of EPR imaging to study animals larger than mice and rats. The same procedure of image registration was applied to assess the thiol redox state, specifically the estimation of intracellular thiol concentration in vivo. Epel et al. used (RS)rapid scan EPR imaging to obtain 3D EPR images in very short time, ~30 s [8, 9]. Changes in EPR line of PxSSPx spine probe sensitive to intracellular thiol concentration were acquired on physiologically relevant timescales and illustrated the enabling capability of RS EPR imaging.

**Fig. 4:**
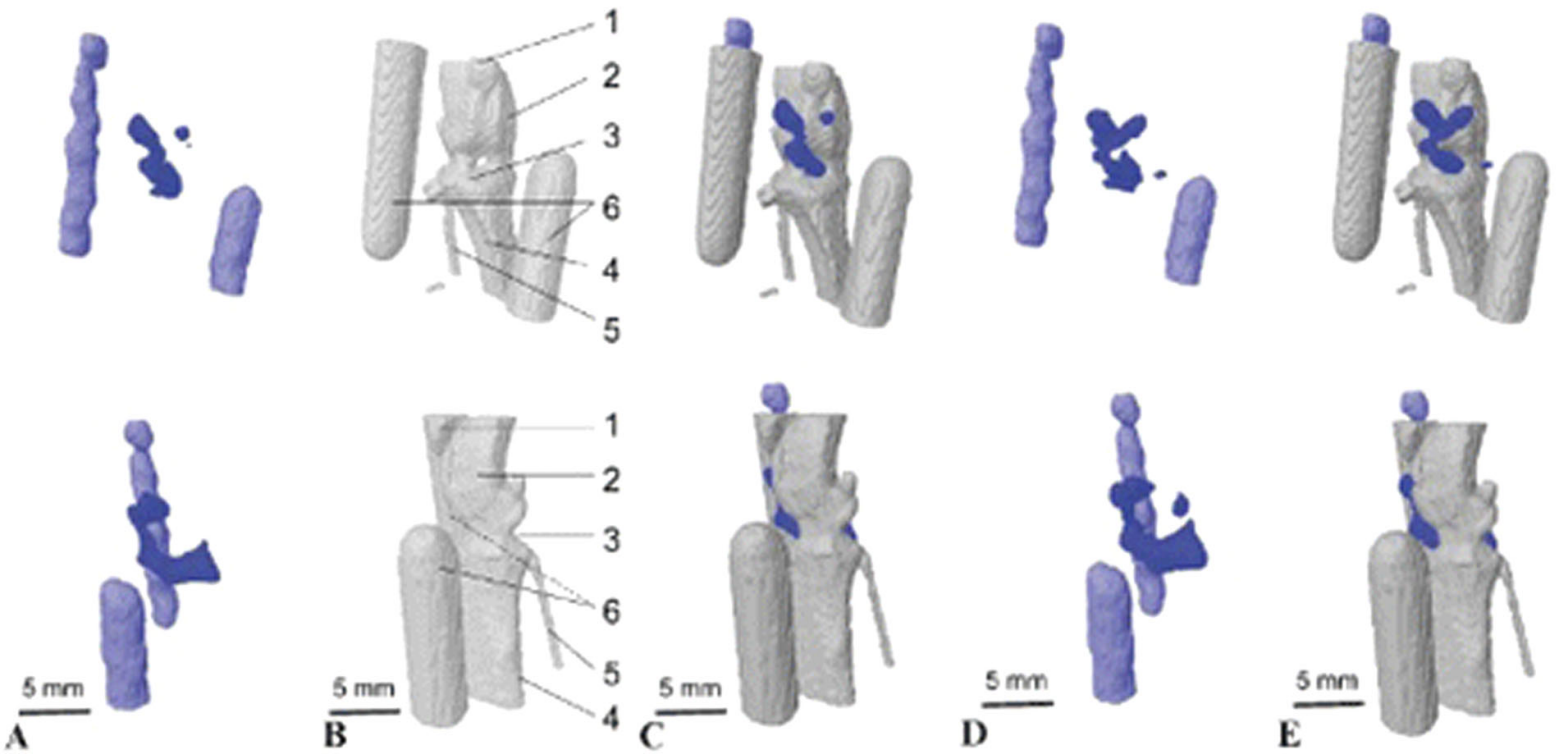
In vivo image registration micro-CT and EPR of mouse knee joint after intra-articular injection of TAM. Frontal (upper) and medial (lower) views of the raw EPR image of the mouse knee joint and of two glass capillaries containing a TAM solution that were placed on each side of the mouse knee joint to locate it. **b** Anatomic x-ray micro-CT image of the same mouse knee and capillaries. **c** Spatial registration, and fusion of the raw EPR and micro-CT images using capillaries as fiducial points. **d** Molecular EPR image of the mouse knee joint after mathematical reconstruction of the image taking into account TAM disappearance during data acquisition. **e** Spatial registration of image D to the micro-CT anatomic image (**b**) of the same mouse knee. On **b**,1 5 patella; 2 5 femur; 3 5 joint cavity; 4 5 tibia; 5 5 fibula; 6 5 reference capillaries. A small volume of TAM was detected away from the bone on the inner side of the knee (see **d** and **e**). This could be a small drop of radical located on the skin, where the needle was inserted (adapted from [35])

Supporting EPRI by other imaging methods, especially MRI in both hybrid systems and with image registration process provides important and unique information about tumor physiology and prospects for a new range of disease treatments. However, MRI is not the only modality providing the necessary anatomical information. Beziere et al. in 2012 demonstrated image registration of EPRI with micro-CT for the study of the knee joint in the mouse. EPRI was used to measure the intra-articular oxidative stress involved in various pathological situations, including osteoarthritis, and rheumatoid arthritis in the knee joint (Fig. 4, [35]).

Another image registration application taking EPRI into account, more specifically redox state of mice brain, was exhaustively investigated by the Hirata’s group. One example is a correlation of brain MRI with redox state in a brain-diseased mouse model using nitroxides as a spin probe [36]. Further studies relating the use of EPR imaging in vivo with image registration are summarized in Table 2.

**Table 2:** EPRI in vivo studies using co-registration with other imaging techniques

Authors	EPR imaging	Spin probe	Supported technique	In vivo Application
Afeworki et al. [47]	3D (spin probe distribution)	TAM^a^	Fiducials surgically placed in the ROI	Mouse liver and kidney
He et al. [17]	3D (spin probe distribution)	3-CP^b^	MRI	Mouse whole body
Elas et al. [48]	4D (pO_2_ maps)	Ox063	*OxyLite*	Tumor
Hyodo et al. [49]	2D (spin probe distribution)	3-CP	MRI	Mouse heart, liver, and kidneys
Matsumoto et al. [50]	2D (redox status)	3-CP	MRI	Tumor
Elas et al. [31]	4D (pO_2_ maps)	Ox063	MRI	Tumor
Matsumoto et al. [51]	4D (pO_2_ maps)	TAM	MRI	Tumor
Haney et al. [33]	4D (pO_2_ maps)	Ox063	DCE-MRI	Tumor
Epel et al. [34]	4D (pO_2_ maps)	Ox063	MRI	Tumor (rabbit)
Elas et al. [52]	4D (pO_2_ maps)	Ox063	Biopsy Needle	Tumor
Fuji et al. [36]	3D (redox status)	HMP	MRI	Mouse head
Matsumoto et al. [53]	4D (pO_2_ maps)	TAM	13C- MRI	Tumor
Elas et al. [32]	4D (pO_2_ maps)	Ox063	MRI	Tumor
Takakusagi et al. [54]	(pO_2_ maps)	Ox063	MRI	Tumor
Radler et al. [55]	3D (spin probe distribution)	Nitroxides^c^, Ox063	MRI	Tumor
Neveu et al. [56]	3D (redox status)	Ox063	13C-MRI, PET, CT	Tumor
Epel et al. [9]	3D (intracellular thiol concentration maps)	PxSSPx^d^	MRI	Tumor

^a^Triarylmethyl radicals

^b^3-Carbamoyl-proxyl

^c^cis-3,4-Di(acetoxymethoxycarbonyl)-2,2,5,5-tetramethyl-1- pyrrolidinyloxyl, cis-3,4-dicarboxy-2,2,5,5-tetra- methyl-1-pyrroldinyloxyl

^d^disulfide-dinitroxide

### Registration Using Features of the Images

Functional images, such as oxygen images may not exhibit sufficient features for precise registration. Therefore, the application of algorithmic feature-based registration such as entropy-based registration may not be practical. Similar functional images, however, may exhibit sufficient common information. In addition to the information provided by the images, one could use a priori information such as animal anatomic details, organ localization, approximate orientations of the object in the imagers, etc. Even if such registration is rather unprecise, it is worthwhile to present EPRI images in anatomical or structural context. Many significant biomedical studies have been performed using approximate registrations. E.g., Williams et al. have shown consistency between EPRI pO_2_ maps of tumors and BOLD MRI [37]. Sonta et al. demonstrated an increased oxidative stress in the kidneys of diabetic mice by comparison of EPRI studies with immunohistological staining [38]. Further important work where EPRI pO_2_ imaging was compared with other hypoxia-imaging techniques such as PET, OxyLite, or ^19^F-MRI were performed by Tran et al. in 2012 and showed a good correlation between the tested methods [39]. Oxidative stress study performed ex vivo in human biopsies was reported by Gustafsson et al. They presented the distribution of oxidative stress of atherosclerotic plaques mapped using EPRI of ROS sensitive spine probe and compared it against histopathology images [14]. Significant examples where EPRI has been compared with other imaging techniques without utilizing hybrid systems or the registration procedure are shown in Table 3.

**Table 3:** Examples of in vivo EPRI studies supported with other imaging techniques using visual image comparison

Authors	EPR imaging	Spin probe	Supported technique	In vivo application
Yokoyama et al. [16]	2D (spin probe distribution)	ACP^a^	Brain atlas	Mouse head
Sano et al. [15]	2D (spin probe distribution)	CxP^b^	Brain atlas	Mouse head
Williams et al. [37]	2D (spin probe distribution)	TAM	MRI (BOLD)	Tumor
Yokoyama et al. [57]	2D (spin probe distribution)	HMP^c^, PCAM^d^	Brain atlas	Mouse head
Velayutham et al. [58]	3D (spin probe distribution)	3-CP	Histology	Mouse heart
Utsumi et al. [59]	2D (spin probe distribution)	MC-PROXYL^e^, AMC-PROXYL^f^, 3-CP	Brain atlas	Mouse head
Ilangovan et al. [60]	2D (spin probe distribution) and 2DS (pO_2_ maps)	LiPc^g^	MRI	Tumor
Sonta et al. [38]	2D (spin probe distribution)	3-CP	Immunostaining	Mouse kidney
Sato-Akaba et al. [13]	3D (spin probe distribution)	TEMPOL-d17,–15N^h^, HMP	MRI	Mouse head
Fuji et al. [36]	3D (redox status)	HMP	MRI	Mouse head
Emoto et al. [61]	3D (spin probe distribution)	HMP, 3-CP, TEMPONE^i^	MRI	Mouse head
Godechal et al. [62]	3D (spin probe distribution)	Melanin	Bioluminescence	Mouse lungs
Tran et al. [39]	(pO_2_ maps)	charcoal wood power	19-FMRI, PET, *OxyLite*	Tumor
Emoto et al. [63]	3D (redox status)	HMP, MCP^j^	MRI	Mouse head
Gustafsson et al. [14]	2D (spin probe distribution)	CMH^k^	Histopathology	Atherosclerotic plaque
Emoto et al. [6]	3D (spin probe distribution)	MCP	MRI	Mouse head

^a^1-acetoxy-3-car- bamoyl-2,2,5,5-tetramethylpyrrolidine

^b^3-carboxy-2,2,5,5-tetramethyl-pyrroli- dine-1-oxyl

^c^3-Hydroxymethyl-2,2,5,5-tetramethylpyrrolidine-1-oxyl

^d^3-methoxycarbonyl-2,2,5,5-tetramethylpyrrolidine-1-oxyl

^e^Carboxy-proxyl methyl ester

^f^Acetyoxymethyl ester of carboxy-proxyl

^g^Lithium phthalocyanine

^h^4-Hydroxy-2,2,6,6-tetramethylpiperidine-d17-1-15N-1-oxyl

^i^2,2,6,6-tetraethyl-4-piperidone-N-oxyl

^j^3-Methoxycarbonyl-2,2,5,5-tetramethylpyrrolidine-1-yloxy

^k^1-hydroxy-3-methoxycarbonyl-2,2,5,5-tetramethylpyrrolidine

## Conclusions

Merging other imaging techniques with EPR imaging seems to be beneficial both for human and preclinical studies. Functional information from EPR imaging supplemented with anatomical knowledge delivers information about oxygenation, redox state, pH, or thiol concentration to be obtained from a chosen ROI. It is crucial that such information is precisely located in multidimensional anatomical space, so that all its aspects—spatial, spectral, and temporal can be correctly interpreted. Thanks to the continuous development of EPR methods, software with a user-friendly interface, combining EPR devices with other systems and the synthesis of advanced spine probes, these solutions enable obtaining many significant results in both human and preclinical biomedical studies.

## References

[1] Epel B, Sundramoorthy SV, Mailer C, Halpern HJ (2008). A versatile high speed 250 MHz pulse imager for biomedical applications. Concepts in Magnetic Resonance Part B Magnetic Resonance Eng..

[2] Ahmad R, Samouilov A, Zweier JL (2016). Accelerated dynamic EPR imaging using fast acquisition and compressive recovery. Journal of Magnetic Resonance.

[3] Sato-Akaba H, Kuwahara Y, Fujii H, Hirata H (2009). Half-life mapping of nitroxyl radicals with three-dimensional electron paramagnetic resonance imaging at an interval of 3.6 seconds. Analytical Chemistry.

[4] Czechowski T, Chlewicki W, Baranowski M, Jurga K, Szczepanik P, Szulc P, Tadyszak K, Kedzia P, Szostak M, Malinowski P, Wosinski S, Prukala W, Jurga J (2014). Two-dimensional EPR imaging with the rapid scan and rotated magnetic field gradient. Journal of Magnetic Resonance.

[5] Eaton SS, Shi Y, Woodcock L, Buchanan LA, McPeak J, Quine RW, Rinard GA, Epel B, Halpern HJ, Eaton GR (2017). Rapid-scan EPR imaging. Journal of Magnetic Resonance.

[6] Emoto MC, Matsuoka Y, Yamada K, Sato-Akaba H, Fujii HG (2017). Non-invasive imaging of the levels and effects of glutathione on the redox status of mouse brain using electron paramagnetic resonance imaging. Biochemical and Biophysical Research Communications.

[7] Gorodetsky AA, Kirilyuk IA, Khramtsov VV, Komarov DA (2016). Functional electron paramagnetic resonance imaging of ischemic rat heart: monitoring of tissue oxygenation and pH. Magnetic Resonance in Medicine.

[8] Epel B, Maggio M, Pelizzari C, Halpern HJ (2017). Electron paramagnetic resonance pO2 image tumor oxygen-guided radiation therapy optimization. Advances in Experimental Medicine Biology.

[9] Epel B, Sundramoorthy SV, Krzykawska-Serda M, Maggio MC, Tseytlin M, Eaton GR, Eaton SS, Rosen GM, Kao JPY, Halpern HJ (2017). Imaging thiol redox status in murine tumors in vivo with rapid-scan electron paramagnetic resonance. Journal of Magnetic Resonance.

[10] Lurie DJ, Li H, Petryakov S, Zweier JL (2002). Development of a PEDRI free-radical imager using a 0.38 T clinical MRI system. Magnetic Resonance in Medicine.

[11] Goshtasby AA (2012). Image Registration. Principles, Tools Methods.

[12] Hirata H, He G, Deng Y, Salikhov I, Petryakov S, Zweier JL (2008). A loop resonator for slice-selective in vivo EPR imaging in rats. Journal of Magnetic Resonance.

[13] Sato-Akaba H, Abe H, Fujii H, Hirata H (2008). Slice-selective images of free radicals in mice with modulated field gradient electron paramagnetic resonance (EPR) imaging. Magnetic Resonance in Medicine.

[14] Gustafsson H, Hallbeck M, Lindgren M, Kolbun N, Jonson M, Engström M, de Muinck E, Zachrisson H (2015). Visualization of oxidative stress in ex vivo biopsies using electron paramagnetic resonance imaging. Magnetic Resonance in Medicine.

[15] Sano H, Naruse M, Matsumoto K, Oi T, Utsumi H (2000). A new nitroxyl-probe with high retention in the brain and its application for brain imaging. Free Radical Biology & Medicine.

[16] Yokoyama H, Itoh O, Aoyama M, Obara H, Ohya H, Kamada H (2000). In vivo EPR imaging by using an acyl-protected hydroxylamine to analyze intracerebral oxidative stress in rats after epileptic seizures. Magnetic Resonance Imaging.

[17] He G, Deng Y, Li H, Kuppusamy P, Zweier JL (2002). EPR/NMR co-imaging for anatomic registration of free-radical images. Magnetic Resonance in Medicine.

[18] Matsumoto S, Nagai M, Yamada K, Hyodo F, Yasukawa K, Muraoka M, Hirata H, Ono M, Utsumi H (2005). A composite resonator assembly suitable for EPR/NMR coregistration imaging. Concepts in Magnetic Resonance Part B Magnetic Resonance Engineering.

[19] Samouilov A, Caia GL, Kesselring E, Petryakov S, Wasowicz T, Zweier JL (2007). Development of a hybrid EPR/NMR coimaging system. Magnetic Resonance in Medicine.

[20] Petryakov S, Samouilov A, Kesselring E, Caia GL, Sun Z, Zweier JL (2010). Dual frequency resonator for 1.2 GHz EPR/16.2 MHz NMR co-imaging. Journal of Magnetic Resonance.

[21] Caia GL, Efimova OV, Velayutham M, El-Mahdy MA, Abdelghany TM, Kesselring E, Petryakov S, Sun Z, Samouilov A, Zweier JL (2012). Organ specific mapping of in vivo redox state in control and cigarette smoke-exposed mice using EPR/NMR co-imaging. Journal of Magnetic Resonance.

[22] Grucker D, Chambron J (1993). Oxygen imaging in perfused hearts by dynamic nuclear polarization. Magnetic Resonance Imaging.

[23] Grucker D, Guiberteau T, Planinsic G (1996). Proton-electron double resonance: spectroscopy and imaging in very low magnetic fields. Research on Chemical Intermediates.

[24] Lurie DJ, Bussell DM, Bell LH, Mallard JR (1988). Proton-electron double magnetic resonance imaging of free radical solutions. Journal of Magnetic Resonance.

[25] Li H, Deng Y, He G, Kuppusamy P, Lurie DJ, Zweier JL (2002). Proton electron double resonance imaging of the in vivo distribution and clearance of a triaryl methyl radical in mice. Magnetic Resonance in Medicine.

[26] Samouilov A, Efimova OV, Bobko AA, Sun Z, Petryakov S, Eubank TD, Trofimov DG, Kirilyuk IA, Grigor'ev IA, Takahashi W, Zweier JL, Khramtsov VV (2014). In vivo proton-electron double-resonance imaging of extracellular tumor pH using an advanced nitroxide probe. Analytical Chemistry.

[27] Kishimoto S, Krishna MC, Khramtsov VV, Utsumi H, Lurie DJ (2018). In vivo application of proton-electron double-resonance imaging. Antioxidants & Redox Signaling.

[28] Pelizzari CA (1998). Image processing in stereotactic planning: volume visualization and image registration. Medical Dosimetry.

[29] Pelizzari CA, Chen GT, Spelbring DR, Weichselbaum RR, Chen CT (1989). Accurate three-dimensional registration of CT, PET, and/or MR images of the brain. Journal of Computer Assisted Tomography.

[30] Haney CR, Fan X, Parasca AD, Karczmar GS, Halpern HJ, Pelizzari CA (2008). Immobilization using dental material casts facilitates accurate serial and multimodality small animal imaging. Concepts in Magnetic Resonance. Part B Magnetic Resonance Engineering.

[31] Elas M, Bell R, Hleihel D, Barth ED, McFaul C, Haney CR, Bielanska J, Pustelny K, Ahn KH, Pelizzari CA, Kocherginsky M, Halpern HJ (2008). Electron paramagnetic resonance oxygen image hypoxic fraction plus radiation dose strongly correlates with tumor cure in FSa fibrosarcomas. International Journal of Radiation Oncology, Biology, Physics.

[32] Elas M, Magwood JM, Butler B, Li C, Wardak R, DeVries R, Barth ED, Epel B, Rubinstein S, Pelizzari CA, Weichselbaum RR, Halpern HJ (2013). EPR oxygen images predict tumor control by a 50% tumor control radiation dose. Cancer Research.

[33] Haney CR, Parasca AD, Fan X, Bell RM, Zamora MA, Karczmar GS, Mauceri HJ, Halpern HJ, Weichselbaum RR, Pelizzari CA (2009). Characterization of response to radiation mediated gene therapy by means of multimodality imaging. Magnetic Resonance in Medicine.

[34] Epel B, Haney CR, Hleihel D, Wardrip C, Barth ED, Halpern HJ (2010). Electron paramagnetic resonance oxygen imaging of a rabbit tumor using localized spin probe delivery. Medical Physics.

[35] Bézière N, Decroos C, Mkhitaryan K, Kish E, Richard F, Bigot-Marchand S, Durand S, Cloppet F, Chauvet C, Corvol MT, Rannou F, Xu-Li Y, Mansuy D, Peyrot F, Frapart YM (2012). First combined in vivo x-ray tomography and high-resolution molecular electron paramagnetic resonance (EPR) imaging of the mouse knee joint taking into account the disappearance kinetics of the EPR probe. Molecular Imaging.

[36] Fujii H, Sato-Akaba H, Kawanishi K, Hirata H (2011). Mapping of redox status in a brain-disease mouse model by three-dimensional EPR imaging. Magnetic Resonance in Medicine.

[37] Williams BB, al Hallaq H, Chandramouli GV, Barth ED, Rivers JN, Lewis M, Galtsev VE, Karczmar GS, Halpern HJ (2002). Imaging spin probe distribution in the tumor of a living mouse with 250 MHz EPR: correlation with BOLD MRI. Magnetic Resonance in Medicine.

[38] Sonta T, Inoguchi T, Matsumoto S, Yasukawa K, Inuo M, Tsubouchi H, Sonoda N, Kobayashi K, Utsumi H, Nawata H (2005). In vivo imaging of oxidative stress in the kidney of diabetic mice and its normalization by angiotensin II type 1 receptor blocker. Biochemical and Biophysical Research Communications.

[39] Tran LB, Bol A, Labar D, Jordan B, Magat J, Mignion L, Grégoire V, Gallez B (2012). Hypoxia imaging with the nitroimidazole 18F-FAZA PET tracer: a comparison with OxyLite, EPR oximetry and 19F-MRI relaxometry. Radiotherapy and Oncology.

[40] Krishna MC, English S, Yamada K, Yoo J, Murugesan R, Devasahayam N, Cook JA, Golman K, Ardenkjaer-Larsen JH, Subramanian S, Mitchell JB (2002). Overhauser enhanced magnetic resonance imaging for tumor oximetry: coregistration of tumor anatomy and tissue oxygen concentration. Proceedings of the National Academy of Sciences of the United States of America.

[41] Li H, He G, Deng Y, Kuppusamy P, Zweier JL (2006). In vivo proton electron double resonance imaging of the distribution and clearance of nitroxide radicals in mice. Magnetic Resonance in Medicine.

[42] Utsumi H, Yamada K, Ichikawa K, Sakai K, Kinoshita Y, Matsumoto S, Nagai M (2006). Simultaneous molecular imaging of redox reactions monitored by Overhauser-enhanced MRI with 14N- and 15N-labeled nitroxyl radicals. Proceedings of the National Academy of Sciences of the United States of America.

[43] Matsumoto S, Yamada K, Hirata H, Yasukawa K, Hyodo F, Ichikawa K, Utsumi H (2007). Advantageous application of a surface coil to EPR irradiation in Overhauser-enhanced MRI. Magnetic Resonance in Medicine.

[44] Matsumoto S, Yasui H, Batra S, Kinoshita Y, Bernardo M, Munasinghe JP, Utsumi H, Choudhuri R, Devasahayam N, Subramanian S, Mitchell JB, Krishna MC (2009). Simultaneous imaging of tumor oxygenation and microvascular permeability using Overhauser enhanced MRI. Proceedings of the National Academy of Sciences of the United States of America.

[45] Ahmad R, Caia G, Potter LC, Petryakov S, Kuppusamy P, Zweier JL (2010). In vivo multisite oximetry using EPR-NMR coimaging. Journal of Magnetic Resonance.

[46] Krishna MC, Matsumoto S, Saito K, Matsuo M, Mitchell JB, Ardenkjaer-Larsen JH (2013). Magnetic resonance imaging of tumor oxygenation and metabolic profile. Acta Oncologica.

[47] Afeworki M, van Dam GM, Devasahayam N, Murugesan R, Cook J, Coffin D, Larsen JH, Mitchell JB, Subramanian S, Krishna MC (2000). Three-dimensional whole body imaging of spin probes in mice by time-domain radiofrequency electron paramagnetic resonance. Magnetic Resonance in Medicine.

[48] Elas M, Ahn KH, Parasca A, Barth ED, Lee D, Haney C, Halpern HJ (2006). Electron paramagnetic resonance oxygen images correlate spatially and quantitatively with Oxylite oxygen measurements. Clinical Cancer Research.

[49] Hyodo F, Yasukawa K, Yamada K, Utsumi H (2006). Spatially resolved time-course studies of free radical reactions with an EPRI/MRI fusion technique. Magnetic Resonance in Medicine.

[50] Matsumoto K, Subramanian S, Devasahayam N, Aravalluvan T, Murugesan R, Cook JA, Mitchell JB, Krishna MC (2006). Electron paramagnetic resonance imaging of tumor hypoxia: enhanced spatial and temporal resolution for in vivo pO2 determination. Magnetic Resonance in Medicine.

[51] Matsumoto, S., Espey, M. G., Utsumi, H., Devasahayam, N., Matsumoto, K., Matsumoto, A., Hirata, H., Wink, D. A., Kuppusamy, P., Subramanian, S., Mitchell, J. B., Krishna, M. C. (2008). Dynamic monitoring of localized tumor oxygenation changes using RF pulsed electron paramagnetic resonance in conscious mice. *Magnetic Resonance in Medicine*, *59*(3), 619–625. 10.1002/mrm.21500. 10.1002/mrm.21500 18224698

[52] Elas M, Hleihel D, Barth ED, Haney CR, Ahn KH, Pelizzari CA, Epel B, Weichselbaum RR, Halpern HJ (2011). Where it's at really matters: in situ in vivo vascular endothelial growth factor spatially correlates with electron paramagnetic resonance pO2 images in tumors of living mice. Molecular Imaging and Biology.

[53] Matsumoto, S., Saito, K., Yasui, H., Morris, H. D., Munasinghe, J. P., Lizak, M., Merkle, H., Ardenkjaer-Larsen, J. H., Choudhuri, R., Devasahayam, N., Subramanian, S., Koretsky, A. P., Mitchell, J. B., Krishna, M. C. (2013). EPR oxygen imaging and hyperpolarized 13C MRI of pyruvate metabolism as noninvasive biomarkers of tumor treatment response to a glycolysis inhibitor 3-bromopyruvate. *Magnetic Resonance in Medicine*, *69*(5), 1443–1450. 10.1002/mrm.24355. 10.1002/mrm.24355 PMC3479339 22692861

[54] Takakusagi Y, Matsumoto S, Saito K, Matsuo M, Kishimoto S, Wojtkowiak JW, DeGraff W, Kesarwala AH, Choudhuri R, Devasahayam N, Subramanian S, Munasinghe JP, Gillies RJ, Mitchell JB, Hart CP, Krishna MC (2014). Pyruvate induces transient tumor hypoxia by enhancing mitochondrial oxygen consumption and potentiates the anti-tumor effect of a hypoxia-activated prodrug TH-302. PLoS ONE.

[55] Redler G, Barth ED, Bauer KS, Kao JP, Rosen GM, Halpern HJ (2014). In vivo electron paramagnetic resonance imaging of differential tumor targeting using cis-3,4-di(acetoxymethoxycarbonyl)-2,2,5,5-tetramethyl-1-pyrrolidinyloxyl. Magnetic Resonance in Medicine.

[56] Neveu MA, De Preter G, Marchand V, Bol A, Brender JR, Saito K, Kishimoto S, Porporato PE, Sonveaux P, Grégoire V, Feron O, Jordan BF, Krishna MC, Gallez B (2016). Multimodality imaging identifies distinct metabolic profiles in vitro and in vivo. Neoplasia (New York, NY).

[57] Yokoyama H, Itoh O, Aoyama M, Obara H, Ohya H, Kamada H (2002). In vivo temporal EPR imaging of the brain of rats by using two types of blood-brain barrier-permeable nitroxide radicals. Magnetic Resonance Imaging.

[58] Velayutham M, Li H, Kuppusamy P, Zweier JL (2003). Mapping ischemic risk region and necrosis in the isolated heart using EPR imaging. Magnetic Resonance in Medicine.

[59] Utsumi H, Yamada K (2003). In vivo electron spin resonance-computed tomography/nitroxyl probe for non-invasive analysis of oxidative injuries. Archives of Biochemistry and Biophysics.

[60] Ilangovan G, Bratasz A, Li H, Schmalbrock P, Zweier JL, Kuppusamy P (2004). In vivo measurement and imaging of tumor oxygenation using coembedded paramagnetic particulates. Magnetic Resonance in Medicine.

[61] Emoto M, Mito F, Yamasaki T, Yamada K, Sato-Akaba H, Hirata H, Fujii H (2011). A novel ascorbic acid-resistant nitroxide in fat emulsion is an efficient brain imaging probe for in vivo EPR imaging of mouse. Free Radical Research.

[62] Godechal Q, Defresne F, Danhier P, Leveque P, Porporato PE, Sonveaux P, Baurain JF, Feron O, Gallez B (2011). Assessment of melanoma extent and melanoma metastases invasion using electron paramagnetic resonance and bioluminescence imaging. Contrast Media & Molecular Imaging.

[63] Emoto MC, Sato-Akaba H, Hirata H, Fujii HG (2014). Dynamic changes in the distribution and time course of blood-brain barrier-permeative nitroxides in the mouse head with EPR imaging: visualization of blood flow in a mouse model of ischemia. Free Radical Biology & Medicine.

